# TGFBIp mediates lymphatic sprouting in corneal lymphangiogenesis

**DOI:** 10.1111/jcmm.14633

**Published:** 2019-08-28

**Authors:** Tong Lin, Xiaozhao Zhang, Yang Lu, Lan Gong

**Affiliations:** ^1^ Department of Ophthalmology Eye, Ear, Nose, and Throat Hospital of Fudan University Shanghai China; ^2^ Laboratory of Myopia NHC Key Laboratory of Myopia (Fudan University) Chinese Academy of Medical Sciences Shanghai China; ^3^ Shanghai Key Laboratory of Visual Impairment and Restoration Fudan University Shanghai China

**Keywords:** inflammation, integrin, lymphangiogenesis, TGF‐β‐induced protein

## Abstract

Corneal lymphangiogenesis plays a key role in diverse pathological conditions of the eye. Here, we demonstrate that a versatile extracellular matrix protein, transforming growth factor‐β induced protein (TGFBIp), promotes lymphatic sprouting in corneal lymphangiogenesis. TGFBIp is highly up‐regulated in inflamed mouse corneas. Immunolocalization of TGFBIp is detected in infiltrating macrophages in inflamed mouse corneas. Subconjunctival injection of liposomal clodronate can significantly reduce macrophage infiltration in inflamed mouse cornea, and decrease the expression of TGFBIp and areas of corneal lymphangiogenesis and angiogenesis after corneal suture placement. In brief, these results indicate that the up‐regulation of TGFBIp in sutured cornea correlates with macrophage infiltration. Although TGFBIp alone cannot significantly stimulate corneal lymph vessel ingrowth in vivo, it can enhance the effect of vascular endothelial growth factor‐C in promoting corneal lymphangiogenesis. The in vitro results show that TGFBIp promotes migration, tube formation and adhesion of human lymphatic endothelial cells (HLECs), but it has no effect on HLECs' proliferation. We also find that the in vitro effect of TGFBIp is mediated by the integrin α5β1‐FAK pathway. Additionally, integrin α5β1 blockade can significantly inhibit lymphatic sprouting induced by TGFBIp. Taken together, these findings reveal a new molecular mechanism of lymphangiogenesis in which the TGFBIp‐integrin pathways plays a pivotal role in lymphatic sprouting.

## 1 INTRODUCTION

Lymphatic system is physiologically essential for the preservation of the isohydria, nutrient uptake and immune surveillance. However, pathological corneal lymphangiogenesis (LG) is involved with diverse eye diseases including dry eye, transplant rejection, herpetic keratitis and ocular allergy.^1^, ^2^, ^3^, ^4^ In fact, recent studies have suggested that corneal LG rather than angiogenesis primarily mediates immune rejection after transplantation.^5^, ^6^ Vision rehabilitating with transplants is threatened by corneal LG because of a high rejection rate ranging from 50% to 90%, in spite of the aggressive treatments.^7^, ^8^, ^9^ Unfortunately, many blind patients caused by corneal diseases are defined as high‐risk transplantation category. Further investigation for the molecular mechanisms of corneal LG may develop new treatment strategies for LG‐related eye diseases.

Although the roles of several growth factors in corneal lymphangiogenesis have been extensively researched, including vascular endothelial growth factor‐C (VEGF‐C), fibroblast growth factor‐2 and platelet‐derived growth factor‐BB,^10^, ^11^, ^12^, ^13^ the regulatory role of extracellular matrix (ECM) molecules in corneal lymphangiogenesis has not been widely evaluated. The newly formed lymphatics are closely linked to the ECM environment due to the absence of a basement membrane and pericytes. Thus, recent studies on the molecular regulation of lymphangiogenesis have focused on the interaction between lymphatic endothelial cells (LECs) and the ECM microenvironment. ECM molecules including hyaluronan, integrins, galectin‐8, matrix metalloproteinases and fibronectin (FN) play a pivotal role in lymphangiogenesis process by regulating LECs' proliferation, migration and tube formation.^14^, ^15^, ^16^, ^17^, ^18^

Transforming growth factor‐β‐induced protein (TGFBIp) is widely expressed in vivo as a kind of ECM protein. It is distributed in corneal epithelium physiologically^19^ and has been found to be overexpressed in a variety of tumours and inflammatory diseases, presenting a certain regulatory effect on cell adhesion, migration and differentiation.^20^, ^21^, ^22^, ^23^, ^24^ TGFBIp contains a highly conserved RGD peptide in the C' section. The RGD peptide is also present in the FN domain, which can be identified by integrin and then mediate FN to induce the sprouting and directional migration of LECs^18^ In previous studies, it has been reported that LECs express several subtypes of integrin which participate in the pathological process of corneal lymphangiogenesis.^25^, ^26^ However, the interaction between LECs, TGFBIp and binding‐receptor integrin is not well understood.

Although previous study found that the inhibition of TGFBIp expression reduces tumour lymphangiogenesis,^27^ its precise function in corneal LG remains obscure. In our current study, a suture‐induced corneal lymphangiogenesis model was used to explore TGFBIp expression in the corneal tissue during the process of inflammation‐induced lymphangiogenesis. The corneal micropocket assay was used to investigate its regulatory role in corneal lymphangiogenesis in vivo. Additionally, in vitro assays, including cell proliferation, adhesion, migration and tube formation, were conducted to investigate the effect of TGFBIp on the biological behaviour of human lymphatic endothelial cells (HLECs) and to preliminarily explore the integrin‐mediated signalling pathways stimulated by TGFBIp. Our current study exploring the regulatory role of TGFBIp in inflammatory corneal lymphangiogenesis may provide a new target for inhibiting corneal lymphangiogenesis and thus lay a foundation for the clinical treatment of lymphatic‐associated disorders.

## 2 MATERIALS AND METHODS

### 2.1 Animals

Mice were treated according to the ARVO Statement for the Use of Animals in Ophthalmic and Vision Research and the recommendations of the National Institutes of Health Guide for the Care and Use of Laboratory Animals. All animal procedures were approved by the Institutional Animal Care and Use Committee at the Eye & ENT Hospital of Fudan University. Mice were anaesthetized using a mixture of ketamine and xylazine (50 mg and 10 mg/kg body weight, respectively) for each surgical procedure. Six‐ to eight‐week‐old male C57BL/6 mice (Laboratory Animal Centre of the Chinese Academy of Sciences in Shanghai, China) were used throughout the study.

### 2.2 Cell culture

Primary HLECs were purchased from PromoCell and cultured in endothelial basal medium (EBM; Lonza) supplemented with 10% foetal calf serum (FCS; Biowest) and other supplements, as previously described.^28^ HLECs at passages 4‐8 were used in this study.

### 2.3 Corneal suture placement

**Figure 1:**
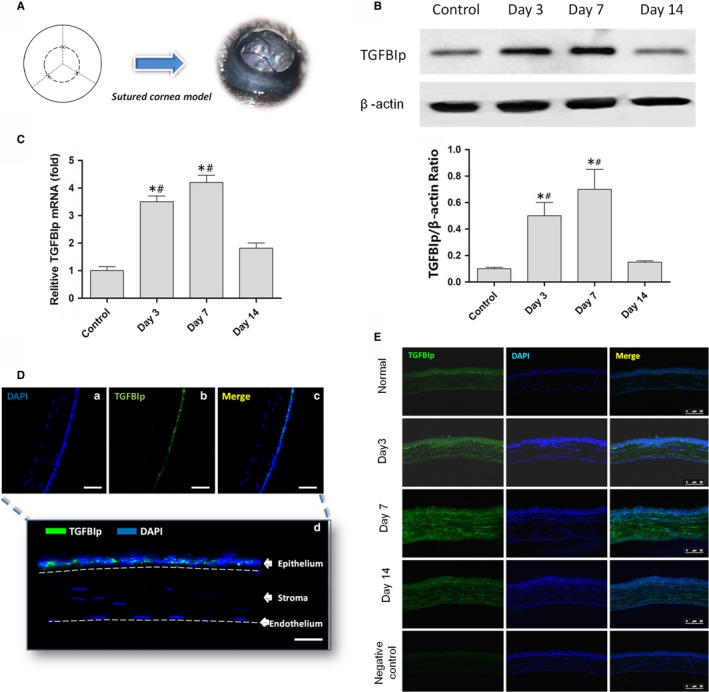
Transforming growth factor‐β induced protein (TGFBIp) is up‐regulated in inflamed mouse corneas. A, The suture model was conducted using C57BL/6 mice. B, The expression change in TGFBIp in mouse cornea by RT‐PCR. C, The expression change in TGFBIp in mouse cornea by the Western blotting assay. D, Immunoreactivity of TGFBIp (green) in normal cornea. Scale bars: 50 μm (a–c); 25 μm (d). (E) Immunoreactivity of TGFBIp (green) in inflamed mouse cornea. Scale bar = 50 μm. *P value < .05 compared with the control, and ^#^ *P* value < .05 compared with day 14

The standard suture placement model was used to induce corneal inflammatory lymphangiogenesis in C57BL/6 mice as described previously.^10^, ^25^ Briefly, three interrupted sutures (11‐0 nylon; Ningbo Medical Needle Co., Ltd.) were placed in the corneal stroma 2 mm away from the limbus without penetrating the anterior chamber to obtain standardized lymphangiogenic responses (Figure 1A).

### 2.4 Mouse corneal micropocket assay and pharmaceutical interventions

The mouse corneal micropocket assay was performed in C57BL/6 mice as previously described.^29^ Test agents included recombinant TGFBIp (40, 80, 160, 320 ng per pellet; R&D Systems) and VEGF‐C (160 ng per pellet; PeproTech). The mouse corneal micropocket assay was surgically created on a single eye of each animal (five mice per group). Implants containing hydron and sucralfate alone served as negative controls. The mice were anaesthetized by intraperitoneal injection of a cocktail of ketamine and xylazine. The eyes were topically anaesthetized with 0.4% oxybuprocaine. Using a corneal blade, intrastromal linear keratotomy was performed approximately 2 mm from the limbus. A pocket was extended towards the limbus using a von Graefe knife, and the pellet was embedded into the pocket. The wound was coated with ofloxacin ophthalmic ointment (Shenyang Xingqi Pharmaceutical Co., Ltd.) to prevent infection. In the combination study, 160 ng TGFBIp and 160 ng VEGF‐C were co‐implanted into each micropocket (five mice per group). For the inhibition study, mice were concurrently injected intraperitoneally with vehicle or integrin α5β1 blocking antibodies (MFR5, 600 μg per mouse; BD Biosciences) on postoperative days 0, 2 and 4. The area of corneal lymphangiogenesis and quantity of lymphatic vessel sprouting were evaluated on day 7 after pellet implantation.

### 2.5 Local depletion of macrophages using subconjunctival clodronate liposomes

Local depletion of macrophages was accomplished as described previously.^30^ Clodronate liposomes (10 μL; Liposoma) was injected subconjunctivally with a microsyringe at the time of suture placement and 2, 4 and 6 days after surgery. Mice in the control group received liposomes containing PBS subconjunctivally at the same time‐points. To confirm whether subconjunctival clodronate liposomes could lead to local depletion of macrophages, immunohistochemistry was performed on corneal whole mounts at 7 days after corneal suture placement for both groups (n = 5 each group) with the macrophage marker (rat antimouse F4/80 antibody; AbD Serotec). Simultaneously, Western blot analysis was conducted to detect the expression change in TGFBIp in the mouse cornea after suture placement in both groups (n = 5 in each group). Additionally, corneal immunofluorescence was conducted with corneal whole mounts to evaluate corneal lymphangiogenesis and haemangiogenesis in both groups (n = 5 in each group).

### 2.6 Quantitative PCR analysis

The quantitative PCR assays were performed to measure the expression levels of TGFBIp and β‐actin. Total RNA was extracted with TRIzol (Invitrogen) from corneal tissue at day 3 (n = 5), day 7 (n = 5) and day 14 (n = 5) after suture placement. The normal corneas (n = 5) were used as the control. RNA was reverse‐transcribed using PrimeScript RT (RR047; Takara) and random primers according to the manufacturer's instructions. Quantitative PCR was performed with a real‐time PCR detection system (Roche LC480) using SYBR Green PCR Master Mix (RR820; Takara) and standard thermocycler conditions. PCR was performed in duplicate in a total volume of 25 μL using 1 μL of cDNA. Each sample was analysed for β‐actin for RNA input amount normalization and to perform relative quantifications. The results are presented with standard deviations. Primer sets synthesized by BioTNT were as follows: β‐actin: 5′‐GGCTGTATTCCCCTCCATCG‐3′ and 5′‐CCAGTTGGTAACAATGCCATGT‐3′; TGFBIp: 5′‐CAGCACGGCCCCAATGTAT‐3′ and 5′‐GGGACCTTTTCATATCCAGGACA‐3′.

### 2.7 Western blot analysis

Western blot analysis was conducted to detect the expression change in TGFBIp in the mouse cornea at day 3 (n = 5), day 7 (n = 5) and day 14 (n = 5) after suture placement, and it was also conducted to detect the expression change in TGFBIp in sutured mouse cornea at day 7 (n = 5) after subconjunctival injection of liposomal clodronate. Normal corneas (n = 5) were used as a control. Furthermore, Western blot analysis was conducted to confirm whether the phosphorylation of FAK stimulated by TGFBIp was mediated by integrin α5β1 or α4β1 and to verify the effect of FAK kinase inhibitors (PF‐573228; Selleckchem) on the phosphorylation of FAK stimulated by TGFBIp. Cell or tissue lysates were fractionated by SDS‐PAGE and transferred to PVDF membranes. The blocked membranes were incubated with the appropriate antibody, and the immunoreactive bands were visualized with a chemiluminescent reagent as recommended by Thermo Scientific, Inc The grey values of the immunoreactive bands were quantified using ImageJ software (developed by Wayne Rasband, National Institutes of Health, Bethesda, MD; available at http://rsb.info.nih.gov/ij/index.html).

### 2.8 TGFBIp immunohistochemistry staining of frozen corneal sections

Frozen sections were obtained from the normal corneas (n = 3) and inflamed corneas of C57BL/6 mice at day 3, day 7 and day 14 after suture placement (n = 3 at each time‐point). Tissue sections of normal and inflamed corneas were processed for immunolocalization of TGFBIp using the following procedure. Briefly, frozen sections were dried, rehydrated and incubated with rabbit antimouse‐TGFBIp primary antibody (1:100 in 1% BSA/PBS, 1 h, 37°C; Abcam) and an Alexa Fluor 488 secondary antibody (1:300 in 1% BSA/PBS, 30 minutes, 37°C; Abcam). Sections were subsequently incubated with a 4′,6‐diamidino‐2‐phenylindole solution (DAPI, 1:1000; 5 minutes, 37°C; Sigma‐Aldrich). Images were acquired using a confocal microscope (Leica TCS‐SP8 Confocal Laser Scanning Microscope; Leica).

### 2.9 F4/80 immunohistochemical staining

For characterization of inflammatory cells recruited to the cornea, immunohistochemistry was performed on corneal whole mounts with the macrophage markers F4/80 (AbD Serotec).^31^ For identification of the intracorneal source of the ECM protein‐TGFBIp, immunohistochemistry of both TGFBIp (1:100 dilution; Abcam) and the macrophage markers F4/80 (1:100 dilution; AbD Serotec) mentioned above was performed on whole mounts of cornea at 7 days after corneal suture placement. The whole mounts of cornea were evaluated using confocal microscopy (Leica TCS‐SP8). Macrophage recruitment was also evaluated as previously described.^32^ Briefly, 10 areas (eight from the periphery and two from the centre) of each sample were randomly picked and examined under an epifluorescence microscope, and the total numbers of F4/80‐positive cells were counted throughout the whole thickness of the selected area.

### 2.10 Corneal immunofluorescence assay and quantification

Corneal whole mounts were used to evaluate corneal neovascularization (lymphangiogenesis and angiogenesis) and lymphatic vessel sprouting. Whole‐mount staining was performed according to our previously described methods.^28^ Mice were killed at the planned times, and the eyes were removed and dissected; subsequently, whole‐mounted corneas were fixed in 4% paraformaldehyde overnight at 4°C and blocked in 5% donkey serum albumin (Solarbio) for 1 hour. For double lymphatic vessel endothelial hyaluronan receptor‐1 (LYVE‐1) and CD31 immunostaining, corneas were incubated overnight with polyclonal rabbit antimouse LYVE‐1 (1/200; Abcam) and rat antimouse CD31 (1/100; BD Biosciences) antibodies. Subsequently, they were incubated overnight with Alexa Fluor 488‐coupled donkey anti‐rabbit antibody (1/200; Abcam) and Cy3‐conjugated donkey anti‐rat antibody (1/200; Jackson ImmunoResearch). Flat mounted corneas on a microscope slide with anti‐fade solution (AR1109; Boster) were examined using a confocal microscope (TCS‐SP8; Leica). Images were obtained at 100× magnification and automatically assembled to reconstitute the whole cornea. The area of corneal lymphangiogenesis and angiogenesis was quantified as described previously using ImageJ software to evaluate the coverage area.^10^, ^33^, ^34^ Lymphatic vessel sprouting was evaluated as follows. Briefly, three areas of each sample on the planted side of the cornea were randomly picked and examined under an epifluorescence microscope, and the total numbers of sprouting lymphatic vessel were counted in the selected area.

### 2.11 LEC proliferation assay

The CCK8‐based proliferation assay was used for our study. Briefly, 1 × 10^4^ HLECs were seeded into each well of a 96‐well plate in a total volume of 200 μL of medium containing 2% (vol/vol) FCS for 4‐6 days. The cells were treated with or without rhTGFBIp (1, 5, or 10 μg/mL). Untreated cells were used as negative controls. After incubation at 37°C for 24, 36 or 48 hours, 10 μL of CCK‐8 (5 mg/mL; Dojindo) was added to each well, and samples were further incubated for 2 hours. The absorbance of the purple formazan solution at a wavelength of 450 nm was measured (n = 6 per group).

### 2.12 LEC migration assay

The wound‐healing assay was performed to evaluate the LEC migration ability. Confluent LEC monolayers grown in 6‐well plates were cultured in starvation medium. Subsequently, scratches were made in each well using the same batch of micropipette tips, and the cells were washed to remove debris. Starvation medium supplemented with rhTGFBIp (1, 5 or 10 μg/mL) was then added. For the comparative study, 5 μg/mL rhTGFBIp or recombinant human fibronectin (rhFN) was added. For the blocking experiments, the LECs were pre‐treated with 1 μg/mL mouse anti‐integrin α5 monoclonal antibody (JBS5; Millipore), 1 μg/mL mouse anti‐integrin α4 monoclonal antibody (9F10; Invitrogen), 1 μmol/L FAK inhibitor (PF‐573228; Selleck) or a nonspecific IgG for 30 minutes before starting the migration experiments. Images were captured at 0 and 12 hours after wounding. For the quantitative analysis, five fields per plate were imaged, and the distances between the front lines were measured using ImageJ software (National Institutes of Health). Each assay was repeated three times.

### 2.13 LEC adhesion assay

Cell‐matrix adhesion assays were performed as described previously.^35^ The 96‐well plates were coated overnight (4°C) with 1‐10 μg/mL rhTGFBIp. LECs in adhesion buffer (serum‐free media) were seeded with 10^5^ cells/well in a 100‐μL volume and incubated for 30 minutes at 37°C. After the removal of non‐adherent cells after two washes, adherent cells were measured by DAPI staining and quantified in triplicate by counting adherent cells in five randomly selected fields per well. For the comparative study, 5 μg/mL rhTGFBIp or rhFN was added. For the blocking experiments, the experiments were performed with or without 1 μg/mL mouse anti‐integrin α5β1 monoclonal antibody, 1 μg/mL mouse anti‐integrin α4β1 monoclonal antibody or 1 μmol/L FAK inhibitor. The results are representative of three different experiments in duplicate.

### 2.14 LEC tube formation assay

Tube formation was assayed as previously described.^27^ In brief, 200 μL of Matrigel (BD Biosciences) was added to a 16‐mm‐diameter tissue culture well and allowed to polymerize for 30 minutes at 37°C. After trypsinization, the harvested LECs were re‐suspended in EBM containing rhTGFBIp (1‐10 μg/mL) and plated onto the layer of Matrigel (1 × 10^5^ cells/well). Matrigel cultures were incubated at 37°C and photographed at various time‐points. For the comparative study, 5 μg/mL rhTGFBIp or rhFN was added. For the blocking experiments, 1 μg/mL mouse anti‐integrin α5β1 monoclonal antibody, 1 μg/mL mouse anti‐integrin α4β1 monoclonal antibody, 1 μmol/L FAK inhibitor or a nonspecific IgG was added to pre‐treat the LECs 30 minutes before starting the tube formation experiments. The lengths of tube formation were quantified in triplicate using ImageJ in five randomly selected fields per well.

### 2.15 Statistical analysis

Statistical data were analysed using SPSS 19.0 (SPSS). Results were expressed as means ± SD. ANOVA followed by Bonferroni post‐tests was performed for analysis of three or more groups. Unpaired Student's *t* test (Mann‐Whitney) was used when only two experimental groups were analysed. A value of *P* < .05 was considered as statistically significant.

## 3 RESULTS

### 3.1 TGFBIp is up‐regulated in inflamed mouse corneas

Corneal suture placement (Figure 1A) was conducted to investigate the change in TGFBIp expression in the inflamed mouse cornea. Normal corneas were used as the control. The RT‐PCR and Western blotting were performed to detect the expression change in TGFBIp in mouse cornea at day 3, day 7 and day 14 after suture placement, and it was found that suture placement induced a significant up‐regulation of both TGFBIp mRNA (Figure 1C) and protein (Figure 1B) at days 3 and 7 after surgery. However, the expression level regressed at day 14. Similar to a previous study,^19^ TGFBIp was mainly expressed in the normal corneal epithelial layer, and TGFBIp immunoreactivity was not detected in the stromal matrix of the normal cornea (Figure 1D). However, TGFBIp was localized to the corneal stroma at day 3, and TGFBIp expression was enhanced at day 7 after suture placement (Figure 1E).

### 3.2 The up‐regulation of TGFBIp in sutured cornea correlates with macrophage recruitment

**Figure 2:**
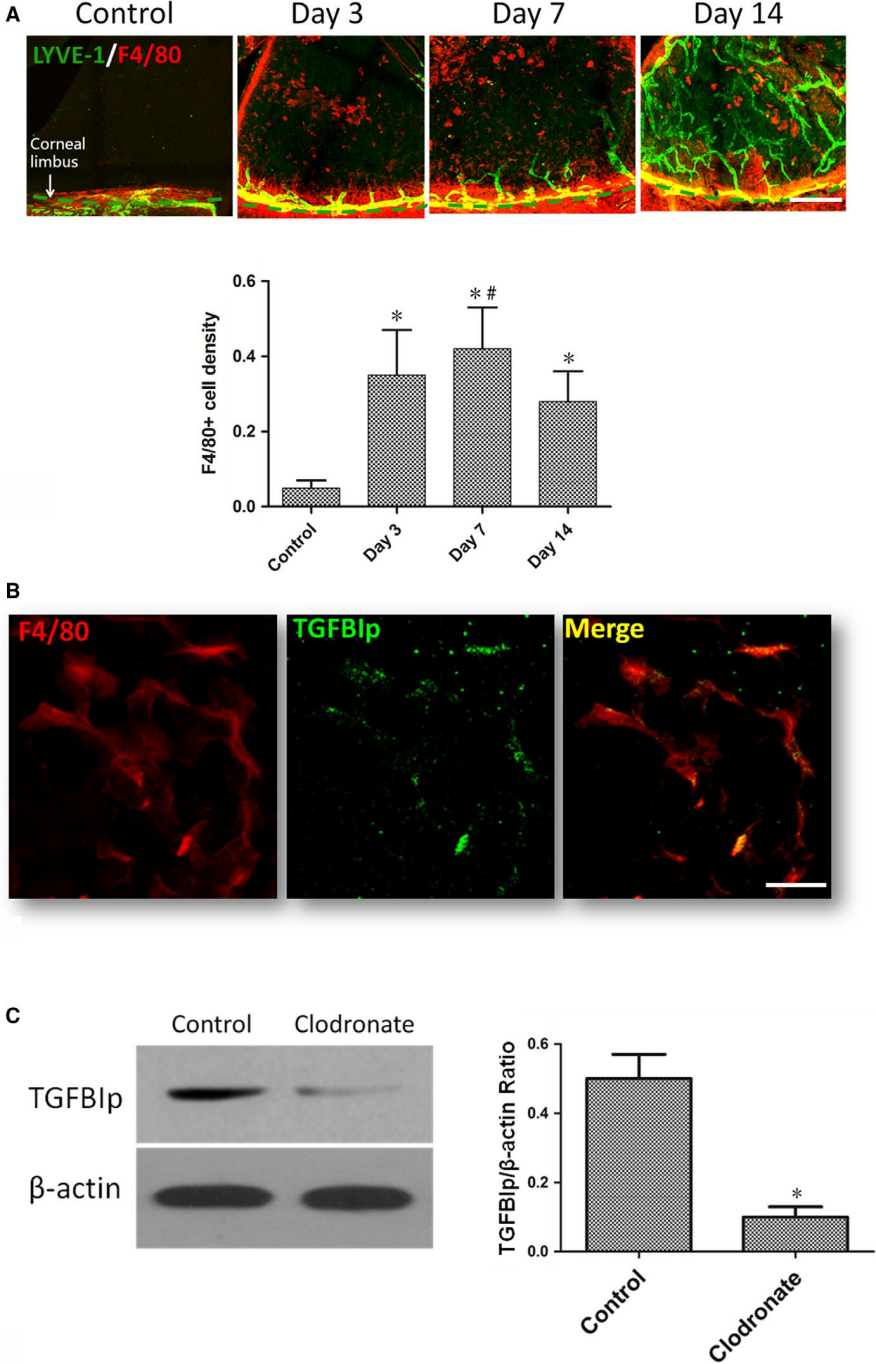
Up‐regulation of transforming growth factor‐β induced protein (TGFBIp) in sutured cornea correlates with macrophage infiltration. A, Corneal suture placement induces both the recruitment of F4/80+ cells (red) and the ingrowth of corneal lymphangiogenesis (green). Scale bar = 400 μm. B, TGFBIp (green) was co‐stained with the macrophage marker F4/80 (red). Scale bar = 20 μm. C, Western blot analysis showed that macrophage elimination resulted in decreased expression of TGFBIp in the sutured cornea. *P value < .05 compared with the control, and ^#^ *P* value < .05 compared with day 14

Previous studies have verified TGFBIp is expressed at significantly elevated levels in macrophages under conditions such as stimulation by inflammation‐associated cytokines^36^ and phagocytosis of apoptotic cells.^37^ F4/80, expressed on the surface of mature tissue macrophages as a 160‐kD glycoprotein,^38^ is commonly used as a macrophage marker. Similar to previous studies,^30^, ^39^ infiltration of abundant F4/80+ cells (macrophages) was detected in mouse corneas after suture placement in our current study. The infiltrated F4/80+ cells increased significantly combined with the ingrowth of corneal lymphangiogenesis at day 3 and day 7 after suture, but the infiltrated F4/80+ cells declined at day 14 and the lymph vessels continued to grow (Figure 2A). Furthermore, to determine whether infiltrated F4/80+ cells expressed TGFBIp in inflamed mouse corneas, vascularized corneas were harvested at 7 days after suture placement, and TGFBIp was co‐stained with the macrophage marker F4/80. The results showed that the expression of TGFBIp co‐localized with F4/80 (Figure 2B). Thus, it is reasonable to suggest that the positively staining cells might represent a possible source of TGFBIp. To verify that the up‐regulation of TGFBIp in sutured cornea correlated with macrophage recruitment, we eliminated the macrophages that infiltrated the sutured cornea via a subconjunctival injection of liposomal clodronate (Figure S1A), which led to decreased expression of TGFBIp (Figure 2C) and reduced areas of corneal lymphangiogenesis and haemangiogenesis at day 7 after suture placement (Figure S1B).

### 3.3 TGFBIp promotes lymphangiogenesis in vitro

**Figure 3:**
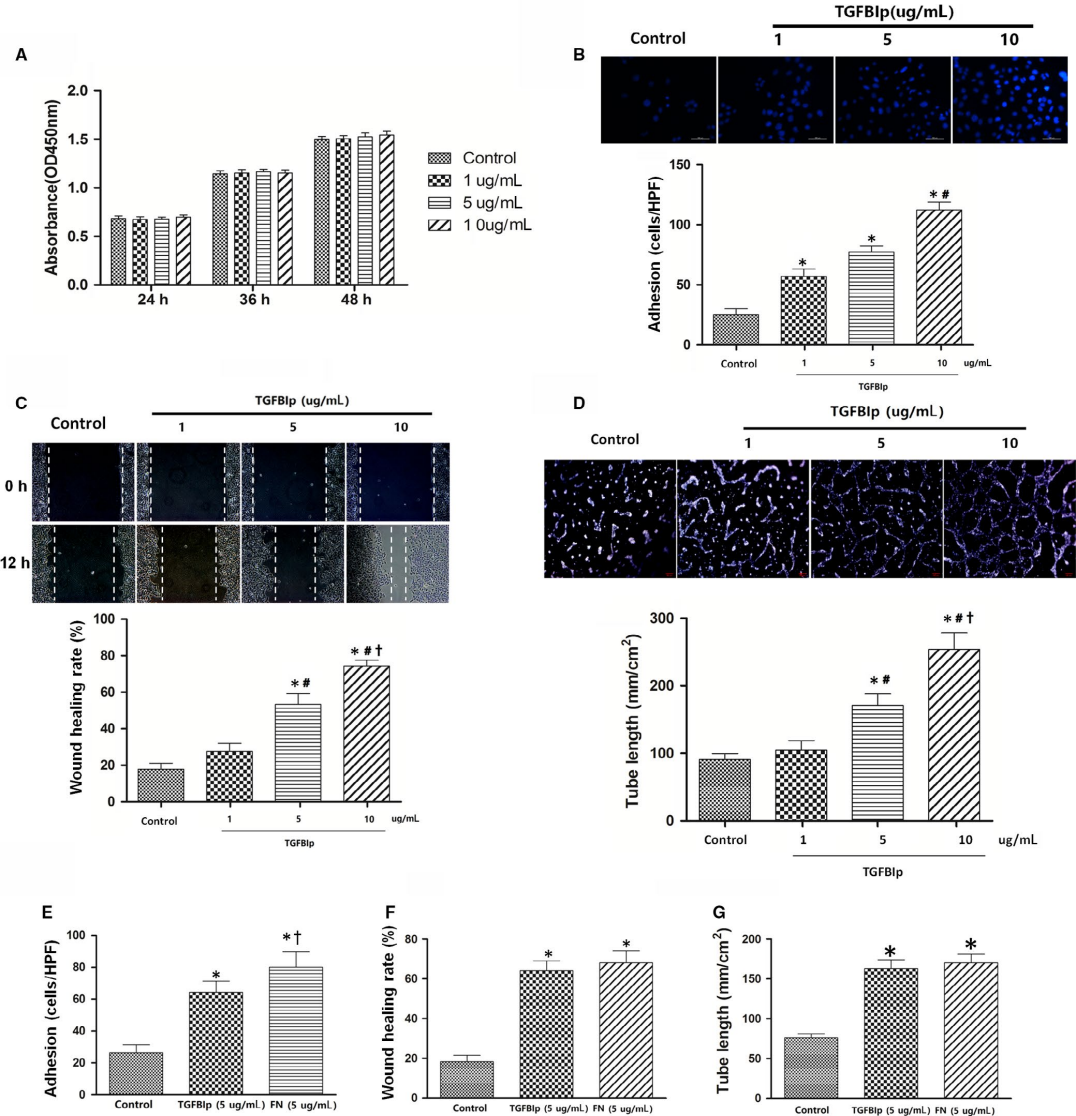
Transforming growth factor‐β induced protein (TGFBIp) promotes lymphangiogenesis in vitro. A, TGFBIp treatment had no effect on human lymphatic endothelial cells' (HLECs) proliferation in vitro. B, The effect of TGFBIp on HLECs' adhesion. Scale bar = 200 μm. C, The effect of TGFBIp on HLECs' migration was evaluated with a scratch‐wound assay. D, TGFBIp treatment promoted HLECs' tube formation in a dose‐dependent manner. E, The differential effect of TGFBIp and fibronectin (FN) on HLECs' adhesion. F, Comparison of the effect on HLECs' migration between TGFBIp and FN. G, Comparison of the effect on HLECs' tube formation between TGFBIp and FN. *P value < .05 compared with the control, ^#^ *P* value < .05 compared with the group with 1 μg/mL TGFBIp, and ^†^ *P* value < .05 compared with the group with 5 μg/mL TGFBIp

To investigate the role of TGFBIp in the regulation of biological behaviour of HLECs in vitro, we observed the effect of TGFBIp on HLECs' proliferation, adhesion, migration and tube formation. Unexpectedly, TGFBIp treatment had no enhancing effect on HLECs' proliferation (Figure 3A). Next, we evaluated the effect of TGFBIp on HLECs' adhesion. The adhesive cells increased significantly in the TGFBIp treatment group compared with the control group, and the promoting effect was dose‐dependent (Figure 3B). We also explored the role of TGFBIp on HLECs' migration with a wound‐healing assay. The uniform scratches were made, and the migration of HLECs into the scratched area was measured 12 hours later. The addition of TGFBIp significantly enhanced HLECs' migration and wound healing in a dose‐dependent manner (Figure 3C). In the next step, we investigated whether TGFBIp has effect on the ability of HLECs' tube formation, and found that TGFBIp treatment also promoted HLECs' tube formation in a dose‐dependent manner (Figure 3D). FN is an important ECM protein consisting of the RGD (Arg‐Gly‐Asp) motif, which is essential for binding integrins that are required for adhesion and migration and tube formation of endothelial cells.^40^, ^41^ Thus, we compared the effects of TGFBIp and FN on adhesion, migration and tube formation in HLECs. Although FN had a superior effect on HLECs' adhesion than TGFBIp (Figure 3E), TGFBIp exhibited a similar enhanced effect on migration and tube formation in HLECs at the same stimulating concentration (Figure 3F,G).

### 3.4 The effect of TGFBIp on lymphangiogenesis in vitro is mediated by the integrin α5β1‐FAK pathway

**Figure 4:**
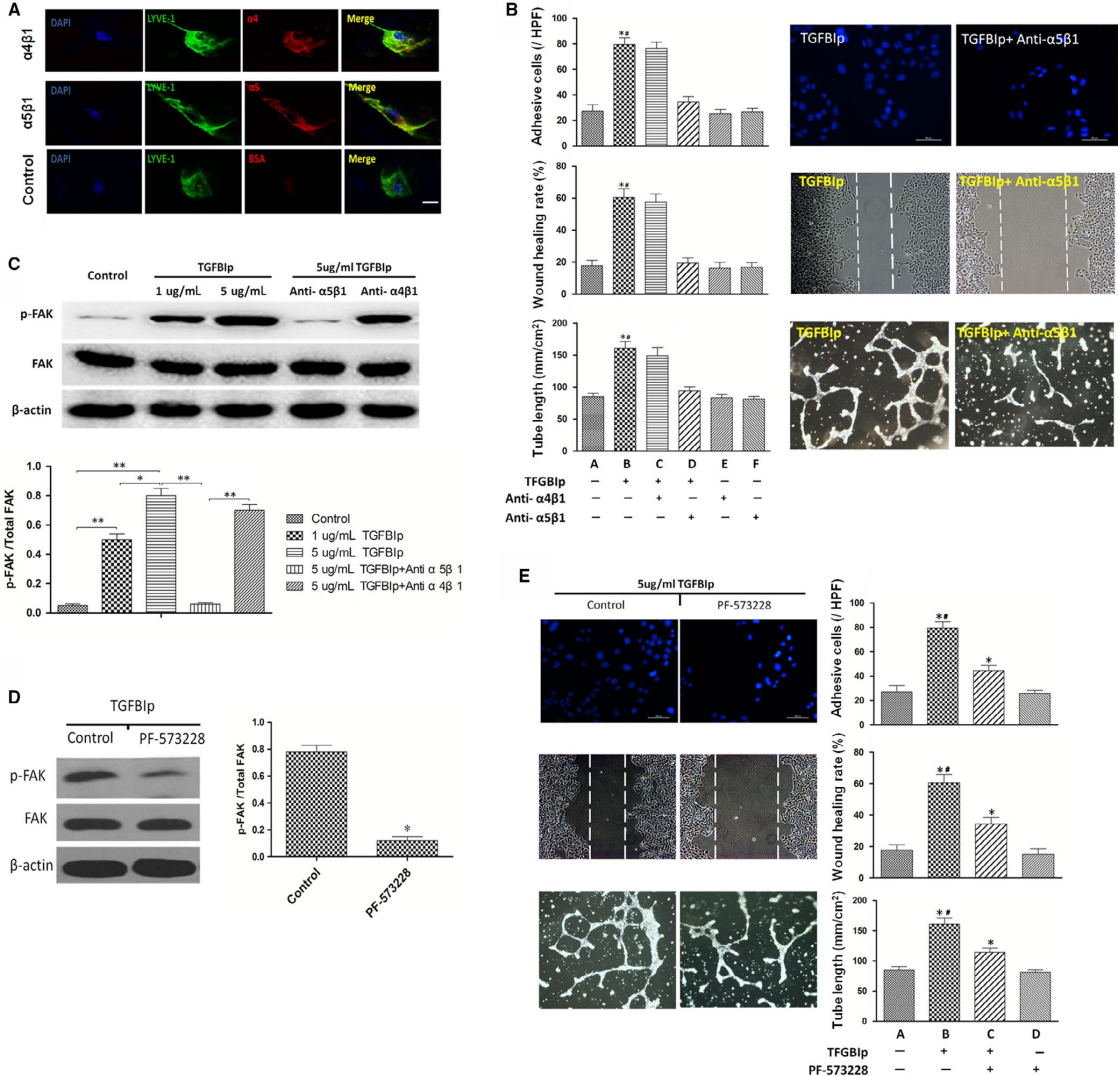
The effect of transforming growth factor‐β induced protein (TGFBIp) on in vitro lymphangiogenesis is mediated by the integrin α5β1‐FAK pathway. A, Integrin α4β1 and α5β1 immunocytochemical staining of primary human lymphatic endothelial cells (HLECs). Scale bar = 10 μm. B, TGFBIp‐induced HLECs' migration, tube formation and adhesion were inhibited by α5β1 integrin blocking antibody, but α4β1 integrin blocking antibody showed few inhibitory effects on cellular function. Scale bar = 200 μm. *P value < .05 compared with group A, and ^#^ *P* value < .05 compared with group D. C, Phosphorylation of FAK stimulated by TGFBIp was suppressed by α5β1 blocking antibodies but not by α4β1 blocking antibodies. *P value < .05, and **P value < .01. D, The inhibitory effect of the FAK kinase inhibitor PF‐573228 on FAK phosphorylation was confirmed by Western blot analysis. *P value < .05 compared with the control. E, The acceleration effects of TGFBIp on HLECs' migration, tube formation and adhesion were partially suppressed by 1 μmol/L PF‐573228. Scale bar = 200 μm. *P value < .05 compared with group A, and ^#^ *P* value < .05 compared with group C

It has been reported that integrins are identified as the cell receptors for TGFBIp^42^, ^43^; however; the subtypes of integrin which mediate the interaction between HLECs and TGFBIp still remain undefined. Using flow cytometry, it was found that HLECs mainly express integrin subtypes including α5β1 and α4β1.^44^ We initially validated that the HLECs used in the current study expressed the integrin subtypes α5β1 and α4β1 using an immunofluorescence assay (Figure 4A). The details of the experimental procedures are provided in Appendix S1. Thus, we sought to elucidate the roles of the integrin subtypes α5β1 and α4β1 in mediating of this important interaction between HLECs and TGFBIp. To this end, we pre‐treated the HLECs with blocking antibody of integrin α4β1 or α5β1, and then conducted an in vitro lymphangiogenesis assay. HLECs' migration, tube formation and adhesion enhanced by TGFBIp were inhibited by integrin α5β1 blocking antibody, but blocking integrin α4β1 showed few inhibitory effects on cellular function as shown in Figure 4B. These results indicate that TGFBIp promotes HLECs' migration, tube formation and adhesion, which could be mediated by integrin α5β1.

The downstream signalling pathways activated by TGFBIp in HLECs need further exploration. Thus, we detected the phosphorylation of possible intracellular signalling molecule FAK when TGFBIp combined with its receptor. Western blotting showed that the phosphorylation of FAK was enhanced by TGFBIp in a dose‐dependent manner. And consistent with the inhibitory effect on cellular function, the phosphorylation of FAK stimulated by TGFBIp was also suppressed by blocking antibodies of integrin α5β1 but not integrin α4β1 (Figure 4C).

To demonstrate whether FAK could mediate the TGFBIp and α5β1 integrin pathway, the FAK kinase inhibitor PF‐573228 was used to investigate its effect on cellular function. First, the inhibitory effect of PF‐573228 on FAK phosphorylation was confirmed by Western blot analysis. Almost 80% phosphorylation of FAK was suppressed by 1 μmol/L PF‐573228 (Figure 4D). Additionally, HLEC migration, tube formation and adhesion enhanced by TGFBIp were partially suppressed by 1 μmol/L PF‐573228 (Figure 4E). These results suggest that the phosphorylation of FAK induced by TGFBIp is mediated by α5β1 integrin.

### 3.5 TGFBIp promotes lymphatic vessel sprouting

**Figure 5:**
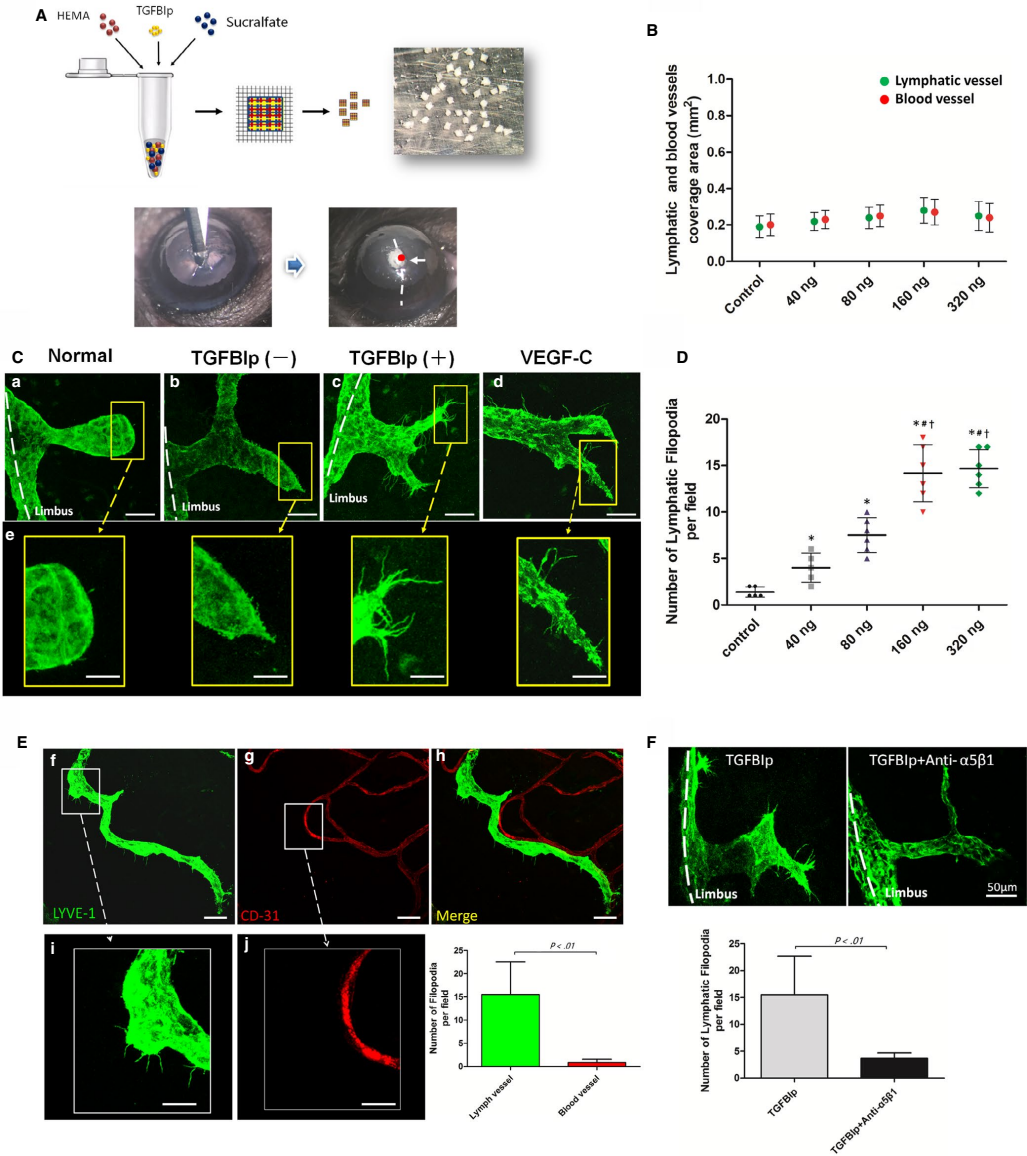
Transforming growth factor‐β induced protein (TGFBIp) promotes lymphatic vessel sprouting. A, Schematic diagram of the corneal micropocket assay. Three key ingredients, TGFBIp, sucralfate and hydron polymer, were well mixed in an Eppendorf tube. The mixture was evenly smeared onto a piece of nylon mesh with an area of 4 mm × 4 mm (approximately 120 grids) and separated into pellets. The clock‐hour position of the circumferential eyeball is indicated. A micropocket was created in the cornea of the right eye of each mouse (labelled the red point). B, TGFBIp could not induce corneal lymph vessel ingrowth. C, Immunolocalization of lymphatic vessel sprouting at the leading front of the limbus lymphatics. Scale bars: 50 μm (a–d); 25 μm (e). D, TGFBIp‐induced tip formation at the leading edge of the lymphatics occurred in a concentration‐dependent manner. E, TGFBIp had no inductive effect on vascular endothelial tips, unlike lymphatic endothelial tips. Scale bars: 50 μm (f–h); 25 μm (i, j). F, MFR5 significantly suppressed lymphatic endothelial tips in the corneal micropocket assay. Scale bars = 50 μm. *P value < .05 compared with the control. ^#^ *P* value < .05 compared with the group with 40 ng TGFBIp, and ^†^ *P* value < .05 compared with the group with 80 ng TGFBIp

The corneal micropocket assay (Figure 5A) showed that TGFBIp could not induce ingrowth of newly formed corneal lymph vessels even at a high dose up to 320 ng (Figure 5B). Previous studies have reported that the formation of endothelial tips is an essential process for the development of newly formed vascular networks.^45^, ^46^, ^47^ Thus, we also analysed the formation of endothelial tips at frontal zone of limbus lymphatics, also termed lymphatic vessel sprouting. Indeed, TGFBIp was able to induce lymphatic endothelial tips at the frontal zone of limbus lymphatics, as the lymphatic endothelial tips in the TGFBIp group were significantly increased compared with the sham control and normal control (Figure 5C). Furthermore, lymphatic vessel sprouting induced by TGFBIp occurred in a concentration‐dependent manner (Figure 5D). Unexpectedly, TGFBIp had no inductive effect on blood endothelial tips, unlike lymphatic endothelial tips (Figure 5E). Next, to determine whether the lymphatic vessel sprouting was mediated by the TGFBIp‐α5β1 integrin pathway, we found that the application of systemically applied integrin α5‐inhibiting blocking antibodies (MFR5) significantly suppressed lymphatic endothelial tips in the corneal micropocket assay compared with the vehicle control (Figure 5F).

### 3.6 TGFBIp and VEGF‐C collaboratively promote corneal lymphangiogenesis

**Figure 6:**
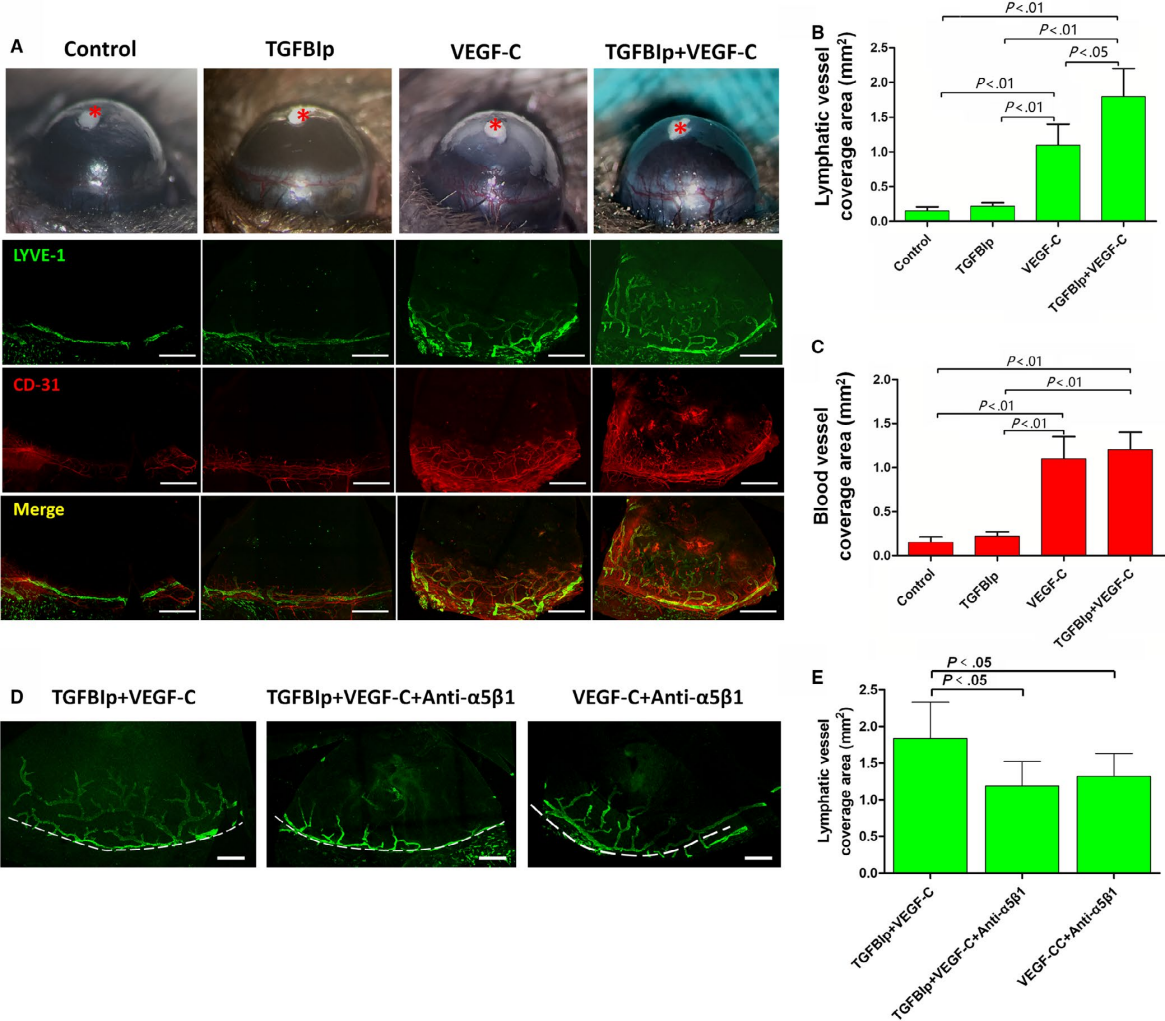
Transforming growth factor‐β induced protein (TGFBIp) and vascular endothelial growth factor‐C (VEGF‐C) collaboratively promote corneal lymphangiogenesis. A, Corneal micropocket assays were conducted to evaluate the synergistic role of TGFBIp and VEGF‐C. LYVE‐1 and CD31 immunostaining were used to evaluate the lymphatic or blood vessel coverage area. Scale bars = 400 μm. B, The lymphatic vessel coverage area was evaluated at 7 d after implanting the micropocket impregnated with nothing, 160 ng of TGFBIp, 160 ng of VEGF‐C or 160 ng of TGFBIp plus 160 ng of VEGF‐C into mouse corneas. C, The blood vessel coverage area was evaluated at 7 d after implanting the micropocket impregnated with nothing, 160 ng of TGFBIp, 160 ng of VEGF‐C or 160 ng of TGFBIp plus 160 ng of VEGF‐C into mouse corneas. D, LYVE‐1 immunostaining was conducted to evaluate corneal lymphangiogenesis for the inhibitory experiment with MFR5. Scale bars = 200 μm. E, The collaborative effect was eliminated by blocking the TGFBIp‐integrin pathway with MFR5

To explore the synergistic effect of TGFBIp on corneal LG in vivo, we implanted uniformed pellets embedded with 160 ng of TGFBIp, 160 ng of VEGF‐C or 160 ng of TGFBIp plus 160 ng of VEGF‐C into the mouse corneas. Hydron pellets without TGFBIp or VEGF‐C were implanted as the sham control. Neovascularization responses of both corneal LG and angiogenesis were quantified by LYVE‐1 and CD31 immunostaining at day 7 after pellet implantation. Pellets with TGFBIp alone did not induce corneal LG, but TGFBIp significantly promoted VEGF‐C‐induced corneal LG as the collaborative effect was stronger than VEGF‐C alone (Figure 6B). Furthermore, the collaborative effect was eliminated by blocking the TGFBIp‐α5β1 integrin pathway with MFR5 (Figure 6D,E).

## 4 DISCUSSION

While most studies on pathogenesis of corneal LG have focused on VEGF‐C and other lymphangiogenic factors,^10^, ^11^, ^12^, ^13^ we demonstrate herein that TGFBIp, a versatile ECM protein, is highly up‐regulated in inflamed mouse corneas and mediates lymphatic sprouting in corneal lymphangiogenesis. Our results also verify that TGFBIp is critically involved in LG processes both in vivo and in vitro assays. Further investigation of this factor may help to develop new therapeutic strategies for LG‐related eye diseases, including dry eye, herpetic keratitis, transplant rejection and ocular allergy.

Previous studies have reported that the formation of endothelial tips is an essential process for the development of newly formed vascular networks.^45^, ^46^, ^47^ A major finding of the current study is that TGFBIp promoted lymphatic sprouting, although TGFBIp alone could not significantly stimulate corneal lymph vessel ingrowth in vivo, which indicated that TGFBIp mainly mediated corneal lymphangiogenesis at the germination stage. The in vitro results showed TGFBIp promoted the migration, tube formation and adhesion of HLECs, but it had no effect on HLEC proliferation, potentially explaining why TGFBIp only promoted lymphatic sprouting but did not induce lymph vessel ingrowth in the mouse corneal micropocket assay. It is possible that TGFBIp plays a regulatory role in the germination stage of lymphatic sprouting but fails to participate in the subsequent stages of lymphatic remodelling and maturation.

Our current study also explored the main source of TGFBIp in inflamed mouse corneas. TGFBIp has been found to be significantly expressed in macrophages under conditions such as stimulation by inflammation‐associated cytokines^36^ and phagocytosis of apoptotic cells.^37^ Consistent with previous studies,^30^, ^39^, ^48^, ^49^ we showed a significant increase in infiltrated macrophages combined with corneal lymphangiogenesis ingrowth. Immunolocalization of TGFBIp was detected in infiltrating macrophages in the inflamed mouse corneas, suggesting that F4/80+ cells may be the possible source of TGFBIp. However, not all macrophages express the same amounts of TGFBIp. We conducted the Western blotting and immunohistochemistry staining to evaluate the TGFBIp expression in different phenotypes of macrophages 3 days after the stimulation of IFN‐γ and IL‐4. In line with the study of Gratchev et al,^36^ the experiments of both immunohistochemistry staining and Western blot analysis presented in Figure S2 demonstrate a strong up‐regulation of the TGFBIp expression in alternatively activated macrophages (M2) by IL‐4 but not in classically activated macrophages (M1) by IFN‐γ when compared with the non‐activated macrophages. Subconjunctival injection of liposomal clodronate has been shown to significantly reduce macrophage infiltration in the inflamed mouse cornea.^30^, ^50^ Our results further showed that macrophage elimination decreased the expression of TGFBIp and areas of corneal lymphangiogenesis and angiogenesis after corneal suture placement. In brief, these results suggested that the up‐regulation of TGFBIp in sutured cornea correlated with macrophage infiltration.

**Figure 7:**
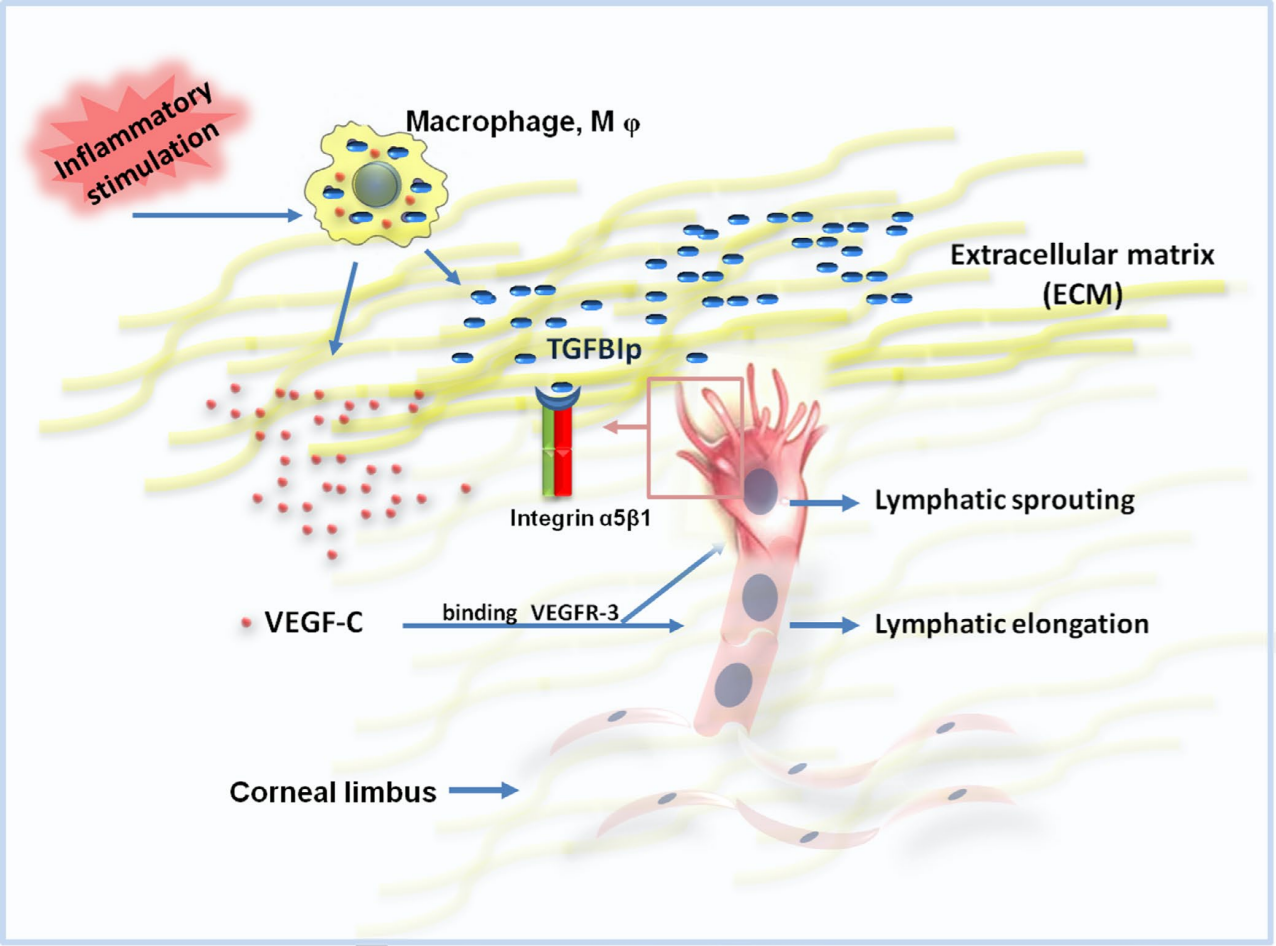
Schematic diagram of molecular mechanisms by which transforming growth factor‐β induced protein and vascular endothelial growth factor‐C (VEGF‐C) collaboratively induce corneal lymphangiogenesis

Another important finding is the synergistic effect of TGFBIp and VEGF‐C in promoting corneal LG. VEGFR‐3 and its ligand VEGF‐C have been proved as a pivotal regulatory signalling axis that induces lymphangiogenesis.^11^ Inflammatory stimulation leads to the infiltration of macrophages, which not only express well‐known lymphangiogenic factor (VEGF‐C) but also give rise to the expression of an ECM protein (TGFBIp). These findings indicate that corneal LG, a complex process, is mediated by both lymphangiogenic factor and ECM molecule. Thus, it is critically essential to develop new therapeutic drugs that interfere with the interplay between diverse lymphangiogenic factors. As shown in Figure 7, TGFBIp and VEGF‐C collaboratively promote lymphangiogenesis in vivo, probably based on TGFBIp‐integrin‐induced LEC tips as a guiding factor and VEGF‐C‐triggered proliferative signals as a paving factor. Thus, TGFBIp could induce tip cell formation, which functions to guide the extension of the newly formed lymph vessel, while VEGF‐C could both promote LEC proliferation and lymphatic sprouting,^11^ which functions to pave the new formed lymph vessel.

We also showed that the in vitro effect of TGFBIp was mediated by the integrin α5β1‐FAK pathway. It has been reported that integrins are identified as the cell receptors for TGFBIp,^51^, ^52^ as TGFBIp contains a highly conserved RGD peptide in the C' section, which can be identified by integrin.^53^ Flow cytometry assessment has shown that HLECs mainly express integrin subtypes, including α5β1 and α4β1.^44^ The downstream signalling pathways activated by TGFBIp in HLECs need further exploration. Thus, we detected the phosphorylation of possible intracellular signalling molecule FAK when TGFBIp combined with its receptor. FAK is the core signalling protein in the integrin downstream signalling pathway, which mediates cell migration and adhesion.^54^, ^55^ Western blotting showed that the phosphorylation of FAK was enhanced by TGFBIp in a dose‐dependent manner and was suppressed significantly by integrin α5β1 blocking but not by α4β1 blockade. Previous studies have demonstrated that integrin α4β1 is more likely to identify Glu‐Ile‐Leu‐Asp‐Val (EILDV) and Arg‐Glu‐Asp‐Val (REDV) than RGD peptide.^56^, ^57^ Furthermore, FAK inhibitor could partially suppress the promoting effects of TGFBIp on HLEC adhesion, migration and tube formation, which indicated that the effect of TGFBIp on in vitro lymphangiogenesis was mediated by FAK to some degree and might also be mediated by other intracellular signalling molecules.

Immunolocalization of integrin α5β1 was detected in the newly formed corneal lymphatic vessels induced by suture placement (Figure S3). The details of the experimental procedures are provided in Appendix S1. The in vivo assay also revealed that systemic delivery of the integrin α5β1 inhibiting antibody (MFR5) could suppress the promoting effect of TGFBIp on lymphatic sprouting and the synergistic effect of TGFBIp and VEGF‐C on corneal LG. These results show that TGFBIp induces the phosphorylation of downstream signalling molecules in pathways by binding to integrin α5β1, which is essential for TGFBIp‐mediated lymphangiogenesis.

And integrin α5β1 blocking provides an alternative strategy to inhibit CL in line with the previous study of Dietrich et al,^58^ which showed integrin α5β1‐specific integrin‐inhibiting small molecule (JSM6427) significantly blocked the outgrowth of new lymphatic vessels into the cornea in a dose‐dependent manner. However, the study of Maeng et al^27^ suggests the lymphatic vessel sprouting stimulated by recombinant TGFBIp is mediated by integrin β3. Thus, the limitation of this study is that our data only showed TGFBIp‐induced lymphatic vessel sprouting was regulated by integrin α5β1. Thus, it still needs further study to verify which integrin isoform plays a more important role in the TGFBIp‐induced lymphatic vessel sprouting.

The current study also reveals a really interesting phenomenon in which TGFBIp plays different roles in corneal LG vs angiogenesis. While lymphatic vessel sprouting was induced by TGFBIp in the corneal micropocket assay, almost no blood vessel sprouting tips were found in the same model. Moreover, TGFBIp and VEGF‐C could not collaboratively promote corneal angiogenesis in vivo. These results indicate that lymphatic vessels are more susceptible than blood vessels to TGFBIp stimulation. The potential mechanisms governing the disparity between LG and angiogenesis responses also need further investigation.

In conclusion, the current study suggests that TGFBIp plays a critical role in corneal LG processes via four probable mechanisms. (a) Inflammatory stimulation leads to the infiltration of macrophages, which not only express the well‐known lymphangiogenic factor VEGF‐C but also TGFBIp. (b) The stimulatory effect of TGFBIp on in vitro lymphangiogenesis is mediated by the integrin α5β1‐FAK pathway. (c) TGFBIp promotes lymphatic sprouting at the germination stage of corneal LG processes. (d) TGFBIp plays a synergistic role with VEGF‐C to promote corneal lymphangiogenesis.

## CONFLICT OF INTEREST

The authors declare no conflict of interest.

## AUTHOR CONTRIBUTIONS

Tong Lin and Lan Gong designed the study. Tong Lin, Xiaozhao Zhang and Yang Lu performed the actual laboratory analyses. Tong Lin, Xiaozhao Zhang and Yang Lu obtained the samples and analysed the data. Tong Lin and Lan Gong wrote and revised the manuscript.

## Data Availability

All the data and materials generated and/or analysed during the current study are available.
