# Supplementary material for: TGFBIp mediates lymphatic sprouting in corneal lymphangiogenesis
Source: J Cell Mol Med. 2019 Aug 28;23(11):7602–16. doi: 10.1111/jcmm.14633 (PMC6815832; doi:10.1111/jcmm.14633)
Supplement: Supplementary file 1 [file JCMM-23-7602-s001.docx]

**Supplementary methods**

**1.Integrin α5 immunohistochemistry staining of newly formed lymphatic vessels**

To confirm whether the newly formed lymphatic vessels in the cornea expressed integrin α5β1, immunohistochemistry was performed on whole mounts of corneas at 7 days after corneal suture placement. Mouse corneas were harvested and fixed in 4% paraformaldehyde overnight at 4°C and blocked in 5% donkey serum albumin (Solarbio) for 1 h. For double lymphatic vessel endothelial hyaluronan receptor-1 (LYVE-1) and integrin α5 immunostain­ing, corneas were incubated overnight with polyclonal rabbit anti-mouse LYVE-1 (1/200; Abcam) and rat anti-mouse integrin α5 (1/100; BD Biosciences) antibodies. Subsequently, they were incubated overnight with Alexa Fluor 488-coupled donkey anti-rabbit antibody (1/200; Abcam) and Cy3-conjugated donkey anti-rat antibody (1/200; Jackson ImmunoResearch). Flat mounted corneas on a microscope slide with antifade solution (AR1109; Boster, Wuhan, China) were examined using a confocal microscope (TCS-SP8, Leica). Images were obtained at 100× magnification and automatically assembled to reconstitute the whole cornea.

**2.Integrin α4 and α5 Immunocytochemical staining**

Primary human lymphatic endothelial cells (HLECs) (1 × 10^5^ cells/well) were seeded onto a round microslide coated with 0.1% polylysine (Sigma-Aldrich), which was placed in a 16-mm diameter tissue culture well for 24 h. Then, the HLECs were fixed in 4% paraformaldehyde overnight at 4°C and blocked in 5% donkey serum albumin (Solarbio) for 1 h. For double lymphatic vessel endothelial hyaluronan receptor-1 (LYVE-1) and integrin α4 or α5 immunostain­ing, the cells were incubated overnight with polyclonal rabbit anti-human LYVE-1 (1/200; Abcam) and mouse anti-human integrin α4 or α5 (1/100; BD Biosciences) antibodies. Subsequently, they were incubated overnight with Alexa Fluor 488-coupled donkey anti-rabbit antibody (1/200; Abcam) and Cy3-conjugated donkey anti-mouse antibody (1/200; Jackson ImmunoResearch). Flat mounted corneas on a microscope slide with antifade solution (AR1109; Boster) were examined using a confocal microscope (TCS-SP8, Leica).
