# Supplementary material for: TGFBIp mediates lymphatic sprouting in corneal lymphangiogenesis
Source: J Cell Mol Med. 2019 Aug 28;23(11):7602–16. doi: 10.1111/jcmm.14633 (PMC6815832; doi:10.1111/jcmm.14633)
Supplement: Supplementary file 2 [file JCMM-23-7602-s002.docx]

**Supplementary figures**


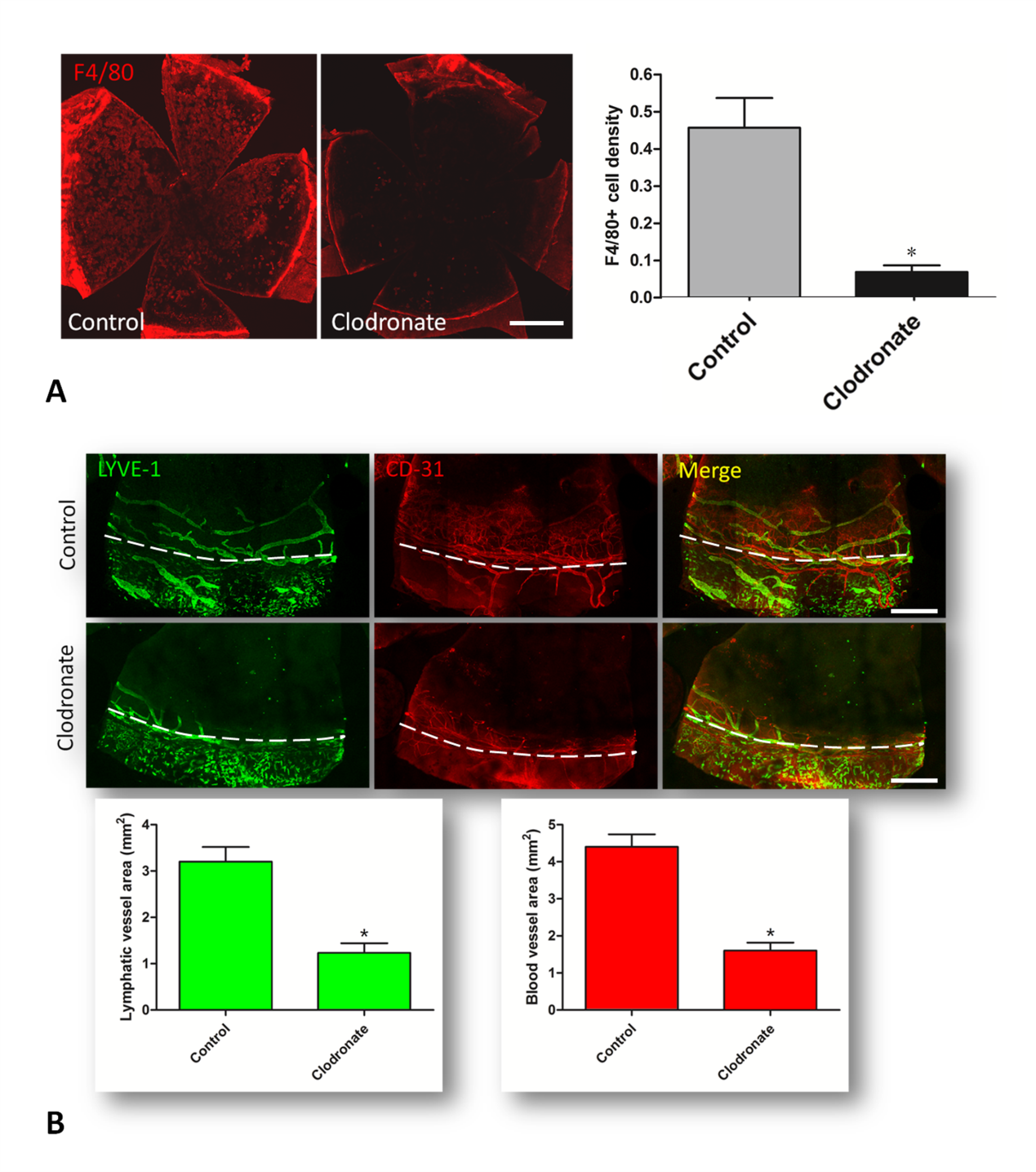


Figure S1. (A) Macrophage recruitment in the sutured cornea was eliminated by subconjunctival injection of liposomal clodronate. Scale bar=800μm (B) Macrophage elimination reduced the areas of corneal lymphangiogenesis and angiogenesis at Day 7 after suture placement. Scale bar=200μm. *indicates P value ＜0.05 compared with the control.

**
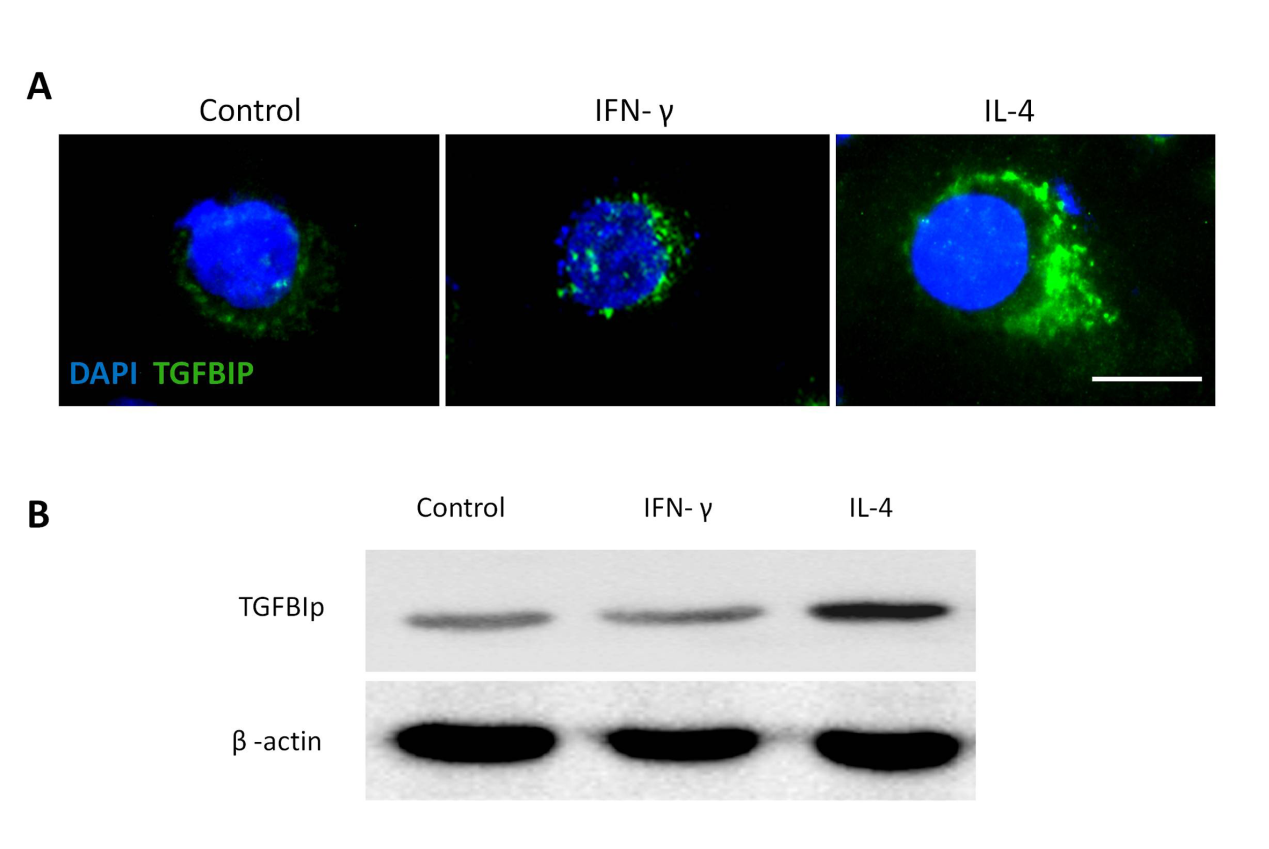
**

Figure S2. Immunohistochemistry staining and Western blot analysis of TGFBIp in macrophages. The experiments of both immunohistochemistry staining (A) and western blot analysis (B) presented here demonstrate a strong upregulation of the TGFBIp expression in alternatively activated macrophages (M2) by IL-4 but not in classically activated macrophages (M1) by IFN-γ. Scale bar=20μm.

**
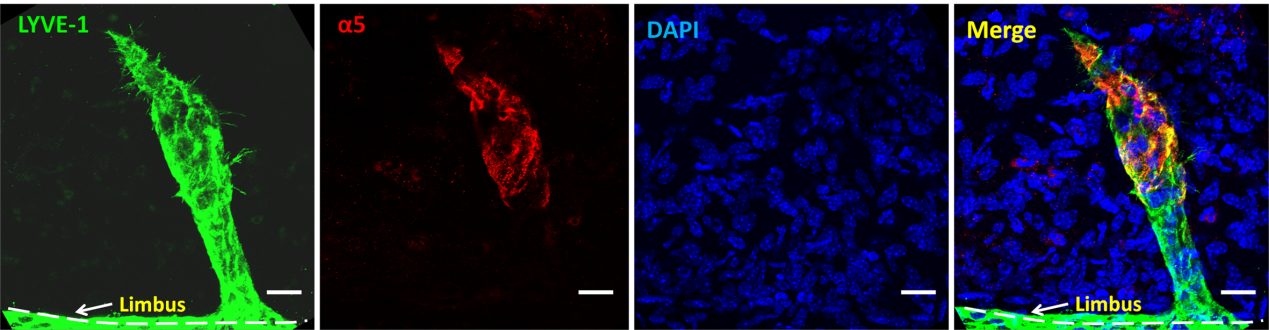
**

**Figure S3.** Immunolocalization of integrin α5β1 was detected in the new formed corneal lymphatic vessels induced by suture placement. Scale bar= 25μm.


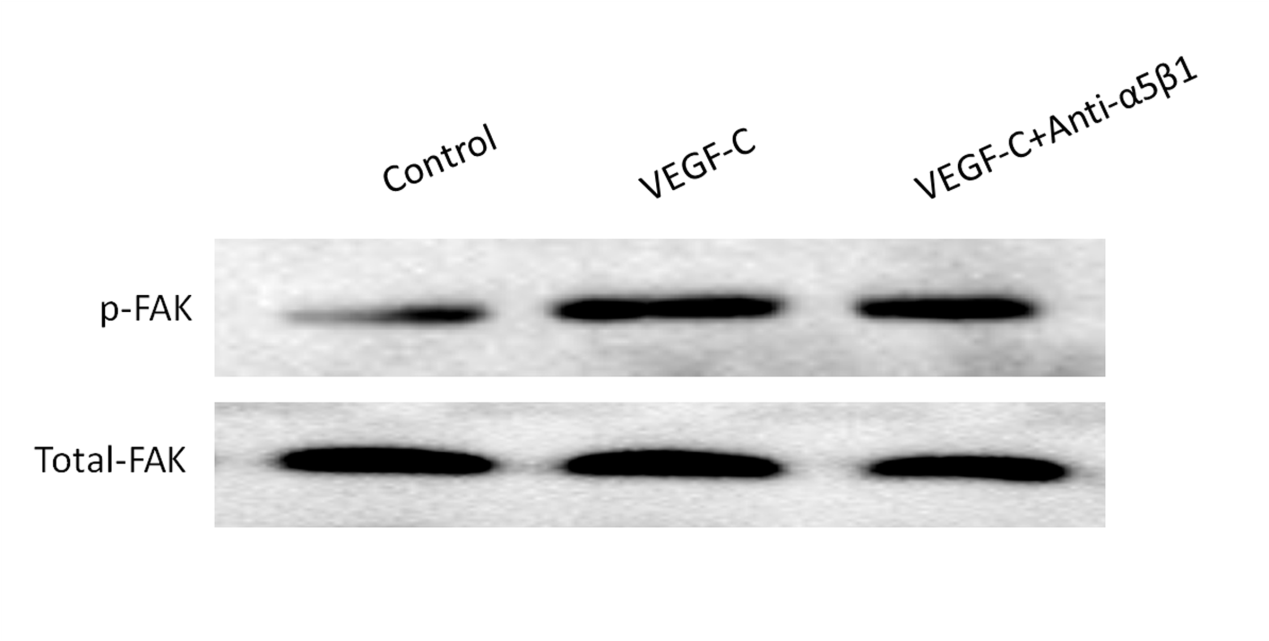


Figure S4. VEGF-C alone stimulated the phosphorylation of FAK in lymphatic endothelial cells, which was not suppressed by integrin α5β1 blocking antibody (JBS5).
